# Housing rental prices: Data from a central urban area of Naples (Italy)

**DOI:** 10.1016/j.dib.2018.03.121

**Published:** 2018-03-30

**Authors:** Vincenzo Del Giudice, Pierfrancesco De Paola, Fabiana Forte

**Affiliations:** a Department of Industrial Engineering, University of Naples “Federico II”, Piazzale Vincenzo Tecchio 80, 80125 Naples, Italy; b Department of Architecture and Industrial Design, University of Campania “Luigi Vanvitelli”, San Lorenzo 31, 81031 Aversa, Italy

## Abstract

The database presented was collected to analyze the housing rental prices for a central urban area of Naples (Italy) during 2016. The data sample relates to 64 housing units located in “Santa Lucia” and “Riviera di Chiaia” neighborhoods. It provides significant information on the urban structure of these neighborhoods. The variables are indicators elaborated from official data sources: real estate rental price, commercial area, maintenance status, number of floor level of housing unit, geographical position. The geographical position is expressed assigning a prefixed sequence of characters to each housing unit, so as to classify every housing unit as falling to a defined sub-area with homogeneous values. The urban area considered is subdivided in five sub-areas. The five subzones are homogeneous in terms of services and infrastructure qualification.

## Specifications

**Table:** 

Subject area	*Economics*
More specific subject area	*Real estate*
Type of data	*Table and graph*
How data was acquired	*Survey*
Data format	*Raw*
Experimental factors	*Sample pretreatment as follows: sources with incomplete data were rejected. The variables surveyed were examined using ordinal or interval scales.*
Experimental features	*First, descriptive statistics were provided, and a correlation matrix was prepared. Then a Multiple Regression Analysis and Genetic Algorithms were used and compared, with the aim to determine the marginal prices of real estate characteristics in order to the formation of real estate rental prices.*
Data source location	*The data were collected from 15 real estate agencies located in the city of Naples.*
Data accessibility	*The data are attached to this article.*

## Value of the data

• The data provides indicators of real estate rental prices (based on official sources) for existing housing units, in a central area in one of the most important Italian cities (Naples). To our knowledge, There are no published databases for the real estate market in Naples, due to the "opacity" – i.e. lack of transparency – of the real estate market (typical condition in Italy), and the scarcity of real estate data freely available.
• The data contains an original indicator – geographical position – constructed (again based on official data sources) to describe the real estate values distribution in the two neighborhoods considered.
• The data presented here can be processed by means of a variety of statistical methods, from multivariate regression to cluster analysis, and hedonic price models.
• To understand the diversity of local real estate values and the relationship between real estate values and real estate characteristic.

## 1 Data

In the field of real estate appraisals often there is low transparency on market information together to stationary conditions [1], [2], these aspects force the analysts to work with single and small dataset for the implementation of hedonic pricing models.

Hedonic price models assume that the values of real estate properties are influenced by their characteristics, with predicted values strongly influenced by dimension and quality of available real estate data. In particular, the minimum dimension of real estate sample necessary to implement a statistical inference model is, as known, correlated to the number of independent variables explaining the real estate characteristics [3].

Therefore, real estate data are essential for the implementation of statistical models used to determine the econometric function of the real estate prices. Hedonic models are widespread in urban studies, as Multiple Regression Analysis or more complex statistical techniques as Genetic Algorithms, Linear Programming, Semi-Parametric or Non Parametric Regressions, Artificial Neural Networks, all these last certainly less commons [3], [4], [5], [6], [7], [8], [9], [10], [11], [12], [13], [14], [15], [16], [17], [18], [19], [20].

The dataset gathers most relevant real estate characteristics for a representative real estate sample related to a central urban area of Naples. The aims is to promote and to make more accessible for scientific and public use a real estate data and unpublished real estate data from the city of Naples. Exclusively available in this open access dataset, there are 576 information gathered in the second semester 2016 related to the housing rental market of two central neighborhoods of Naples.

Real estate data are provided together to an isovalues map with the spatial distribution of real estate rental values for the central area considered. The map provided here may serve as an useful reference for future analysis and application.

From an academic point of view, data can allow experimentation and implementation of inferential statistical models applied to the real estate market. From a managerial point of view, the data are valuable as they allow an immediate perception of the appreciation of the properties, being able to affect future choices of the stakeholders in a very prestigious central area of Naples.

## 2 Experimental design, materials and methods

The data sample relates to 64 housing units located in “Santa Lucia” and “Riviera di Chiaia” neighborhoods in the city of Naples.

Only non-homogeneous real estate characteristics, besides the geographic location, were detected for each sampled unit and, in particular (see Table 1):

• real estate rental price (monthly) expressed in euro (RERP);
• commercial area expressed in square meters (AREA);
• maintenance status (MAIN) expressed with a scores scale: 2 if the housing unit is in optimum conditions, 1 if maintenance status is good, 0 otherwise (mediocre status);
• number of floor level of housing unit (FLOOR);
• geographical position (GEO) expressed with a binary sequence representative of a interval scale (although the geographic coordinates are also provided in the dataset).

**Table 1:** Statistical description of real estate data.

**Variable**	**Std. Dev.**	**Median**	**Mean**	**Min**	**Max**
RERP	6000.00	963.51	1692.00	400.00	6000.00
AREA	71.29	100.00	122.31	30.00	460.00
MAIN	0.63	2.00	1.49	0.00	2.00
FLOOR	1.55	2.00	2.44	0.00	7.00
AREA A	0.25	0.00	0.07	0.00	1.00
AREA B	0.42	0.00	0.22	0.00	1.00
AREA C	0.49	0.00	0.38	0.00	1.00
AREA D	0.43	0.00	0.24	0.00	1.00
AREA E	0.29	0.00	0.09	0.00	1.00

The geographical position (GEO) was expressed assigning a prefixed sequence of characters to each housing unit, so as to classify every housing unit as falling to a defined area among those already identified in a study focused on the city of Naples [4]. The cited study used a Geoadditive Model based on Penalized Spline functions for the housing market segmentation and, specifically, by this work it's possible to subdivide the urban area considered in five subareas with homogeneous values (A, B, C, D, E). The housing units that falling in one of the mentioned five subzones (A, B, C, D, E) are included into a homogeneous urban area in terms of services and infrastructure qualification, in fact, the differences in value for the different sub-areas are small. Sub-areas with highest values are characterized by historical and touristic amenities nearby, while sub-areas with lowest values have almost exclusively a residential vocation.

Even falling in a narrow geographic area, in the D and E zones there is the presence of a great historic castle, many luxury hotels and a pedestrian area. With the same environmental characteristics the housing supply in areas D and E is much more scarce and, consequently, real estate rental prices tend to be higher, although housing units have less fruition ability for parking difficulties, vehicle traffic due to the great presence of tourists, more distance from urban services mainly located in zone C. Instead, the A and B zones have intermediate characteristics respect to the other zones.

**Fig. 1:**
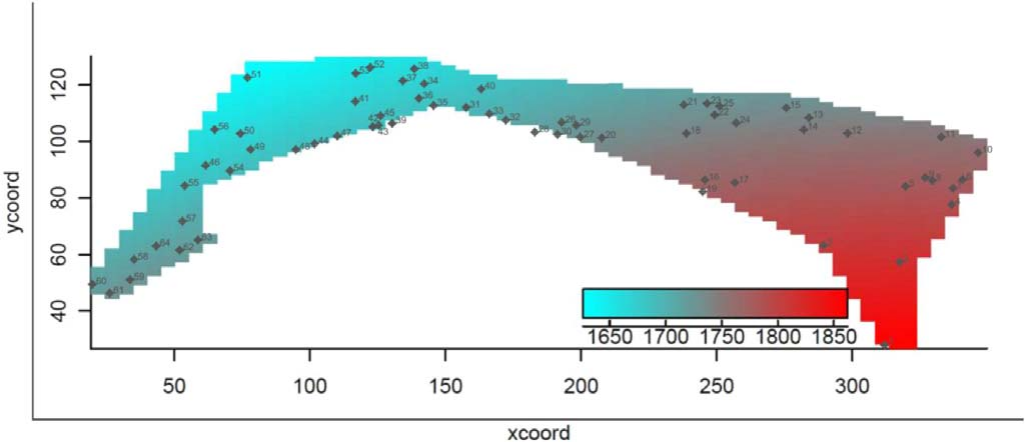
Spatial distribution of real estate rental values [4].

More precisely, mentioned sub-areas were so defined (in terms of monthly rental price; see Fig. 1):

• Area A: mean rental price up to € 1.700,00 (binary sequence: 1,0,0,0,0);
• Area B: mean rental price from € 1.700,00 to € 1.750,00 (binary sequence: 0,1,0,0,0);
• Area C: mean rental price from € 1.750,00 to € 1.800,00 (binary sequence: 0,0,1,0,0);
• Area D: mean rental price from € 1.800,00 to € 1.850,00 (binary sequence: 0,0,0,1,0);
• Area E: mean rental price over € 1.850,00 (binary sequence: 0,0,0,0,1).

The dataset includes, as additional information, also the geographic coordinates of the single housing units sampled (in terms of latitude and longitude).

Other real estate characteristics (as panoramic views, noise, etc.), manifesting with the same modalities in all the sampled units, were excluded from the dataset.

It is worth highlight that housing rental prices data are inadequately collected in Italian research and practice (Naples is not an exception). All the data on housing rental prices and real estate characteristics relate to the second semester 2016.

The data were collected from 15 real estate agencies located in the two neighborhoods surveyed, for this purpose direct interviews were conduct by the authors.

The measurement scales adopted (ordinal and interval scales) are consistent with the literature [21]. The coding systems adopted for the variables were dictated by those available from the sources.

## Funding

This research did not receive any specific grant from funding agencies in the public, commercial, or not-for-profit sector.
